# Selected Demographic and Temperamental Characteristics of Patients with Lung Cancer and Their Associations with Disease Acceptance, Negative Emotional Experiences, and Hope

**DOI:** 10.3390/cancers18213575

**Published:** 2026-11-06

**Authors:** Agnieszka Waleczko, Bożena Baczewska, Beata Barańska, Dagmara Podgórska-Kowalczyk, Krzysztof Leśniewski

**Affiliations:** 1 Department of Internal Medicine and Internal Medicine in Nursing, Faculty of Health Sciences, Medical University of Lublin, Chodźki 7, 20-093 Lublin, Poland; waleczkoagnieszka@gmail.com; 2 Department of Orthopaedic Oncology, Brzozów Specialist Hospital, Subcarpathian Oncology Centre, ul. ks. Bielawskiego 18, 36-200 Brzozów, Poland; bbaranska1@o2.pl; 3 Institute of Health, The State University of Applied Sciences in Przemyśl, ul. Książąt Lubomirskich 6, 37-700 Przemyśl, Poland; d.podgorska-kowalczyk@pansp.pl; 4 Department of Orthodox Theology, Faculty of Theology, The John Paul II Catholic University of Lublin, Al. Racławickie 14, 20-950 Lublin, Poland; lesni@kul.pl

**Keywords:** lung cancer, disease acceptance, Snyder’s Hope Scale, negative emotions, cluster analysis

## Abstract

**Background/Objectives**: A diagnosis of lung cancer can be accompanied by negative emotional experiences. This study aimed to explore relationships between selected sociodemographic and temperamental characteristics, 14 negative emotional states, disease acceptance (AIS), and hope (Snyder’s Hope Scale) in patients with lung cancer. **Methods**: The sample comprised 104 patients with lung cancer (52.88% male, 47.12% female; 57.69% aged 65 years or older). Measures included the AIS, Snyder’s Hope Scale, and an author-developed negative emotions questionnaire. Analyses included Spearman’s rank correlation, non-parametric tests, and exploratory hierarchical cluster analysis (UPGMA). Cluster stability was assessed using 1000 random 80% subsamples; Jaccard distance was used as a sensitivity analysis. **Results**: Most patients (65.39%) reported moderate disease acceptance (mean AIS = 23.14 ± 8.29). Anger (57.69%) and helplessness/powerlessness (40.38%) were the most frequently endorsed emotions. Cluster analysis identified two groups differing primarily in the number and breadth of endorsed negative emotions. Cluster 1 comprised 75% of patients, whereas Cluster 2 comprised 25% and reported a greater variety of emotions. Cluster 2 had higher disease acceptance than Cluster 1 (AIS = 27.19 vs. 21.79; *p* = 0.004). Overall hope (mean = 25.89 ± 5.21) did not differ between clusters (*p* > 0.05). **Conclusions**: Disease acceptance may coexist with a broader range of negative emotional experiences and should not be understood simply as the absence of negative emotions. This exploratory finding requires confirmation using validated measures and longitudinal designs. Dispositional hope did not differ between groups, suggesting that hope and negative emotional experiences may represent distinct dimensions of psychological adaptation.

## Simple Summary

A diagnosis of lung cancer can be accompanied by a wide range of difficult emotions and may affect how patients perceive and accept their illness. This study examined negative emotional experiences, disease acceptance, hope, and selected demographic and temperamental characteristics in 104 patients with lung cancer. The analysis identified two exploratory groups that differed primarily in the number and breadth of endorsed negative emotional experiences. Patients who reported a greater number of negative emotional experiences also showed higher levels of disease acceptance, while hope did not differ significantly between the groups. These findings suggest that a greater number of reported negative emotional experiences does not necessarily indicate poorer psychological adaptation to lung cancer. Disease acceptance, emotional experiences, and hope may represent related but distinct aspects of psychological adaptation. A multidimensional approach to psychological assessment may therefore provide a more complete understanding of patients’ experiences and help identify individual psychosocial needs.

## 1. Introduction

Lung cancer remains the leading cause of cancer-related mortality worldwide and imposes a substantial physical and psychosocial burden on affected individuals [1]. Patients with lung cancer frequently experience substantial psychological distress, with lung cancer patients showing significantly higher levels of anxiety and depressive symptoms than patients with several other cancer types [2,3]. This psychological burden may be further compounded by the high symptom burden and stigma associated with lung cancer [4]. Moreover, poorer quality of life has been associated with an increased risk of mortality among patients with lung cancer [5].

A critical milestone is disease acceptance. Lung cancer patients have been reported to show relatively low levels of disease acceptance compared with patients with other major cancer types [6], while recent evidence indicates that disease acceptance remains an important aspect of psychological adaptation following a lung cancer diagnosis [7]. Disease acceptance has been associated with lower negative affect and better adaptation to physical limitations [6]. To better understand differences in psychological adaptation, it is important to consider demographic and temperamental characteristics. Socio-demographic variables are associated with differences in coping resources and psychological vulnerability [8,9]. Simultaneously, stable personality characteristics, such as neuroticism, may shape psychological adjustment and distress [10,11].

A vital resource is hope. Snyder’s Hope Theory conceptualises hope as a cognitive process consisting of pathways (planning strategies) and agency (goal-directed determination) [12]. Snyder’s Hope Scale offers a validated measure of this goal-directed thinking [12]. In lung cancer, higher hope has been associated with less depression and lower physical symptom severity, including pain, fatigue, and coughing, even after controlling for disease stage [13]. Furthermore, hope is closely related to perceived meaning in life and has been associated with lower levels of depressive and anxiety symptoms [14,15]. More recent evidence in patients with advanced lung cancer similarly suggests that hope is an important factor in understanding psychological distress, including anxiety and depressive symptoms [16].

Temperamental traits are conceptualised in trait psychology and the Regulative Theory of Temperament (RTT) as relatively stable characteristics that may influence individual patterns of behavioural and emotional functioning [17,18]. Under conditions of acute or extreme stress, such as facing a life-threatening illness, such characteristics may be relevant to individual differences in emotional responses and psychological adaptation [17,19].

The novelty of our research lies in examining these variables simultaneously. While existing literature has explored isolated relationships—such as hope and symptoms [13] or disease acceptance and household income [6]—the simultaneous consideration of selected demographic and temperamental characteristics of patients with lung cancer, their disease acceptance, negative emotional experiences, and hope measured using Snyder’s Hope Scale remains insufficiently explored. This approach allowed us to explore whether subgroups of patients characterised by different patterns of reported negative emotional experiences could be identified.

## 2. Materials and Methods

The present study was quantitative, correlational, and cross-sectional in design. Its main aim was to determine the relationships between selected demographic and temperamental characteristics of patients diagnosed with lung cancer and their level of disease acceptance, measured using the Acceptance of Illness Scale (AIS), the intensity of accompanying negative emotions, and the level of hope measured using Snyder’s Hope Scale. The study involved the same cohort of 104 patients as our previous publication [7]. While the previous publication examined disease acceptance and hope using the AIS and Block’s Hope Scale (NCN-36), the present study focuses on reported negative emotional experiences, including their intensity and breadth, and their associations with disease acceptance and hope assessed using Snyder’s Hope Scale.

### 2.1. Research Instruments

A set of structured self-report questionnaires, consisting of established and standardised psychometric methods and an ad hoc survey sheet, was used to gather empirical data.

#### 2.1.1. Acceptance of Illness Scale (AIS)

To measure the degree of adaptation and acceptance of limitations resulting from lung cancer, the Acceptance of Illness Scale (AIS), developed by Felton et al. and adapted into Polish by Zygfryd Juczyński, was used [20,21]. The tool consists of eight statements describing the negative consequences of poor health, including feelings of dependence on others, being a burden, reduced self-esteem, and reduced self-sufficiency. Each statement is rated on a 5-point Likert scale, ranging from 1 (“strongly agree”) to 5 (“strongly disagree”). The total score ranges from 8 to 40 points, with higher scores indicating greater acceptance of illness and better adaptation to the health situation. Scores of 8–18 indicate low acceptance of illness, 19–36 indicate moderate acceptance, and 37–40 indicate high acceptance [21,22]. The Polish version of the scale has demonstrated good internal consistency, with Cronbach’s alpha reported at approximately 0.82–0.85 [21,22].

#### 2.1.2. Snyder’s Hope Scale (Adult Dispositional Hope Scale—ADHS)

The level of patients’ hope as a goal-directed cognitive process was assessed using Snyder’s Adult Dispositional Hope Scale (ADHS), based on Snyder’s theory of hope [23]. The scale consists of 12 items, including 8 diagnostic items and 4 filler items. The diagnostic items assess two interrelated components of hope: agency, referring to goal-directed determination and the perceived ability to initiate and sustain action toward goals, and pathways, referring to the perceived ability to generate alternative routes and strategies for achieving goals. Four items assess agency and four assess pathways, while the remaining four items serve as filler items and are not included in the total score. In the present study, responses were rated on a 4-point scale ranging from 1 (“definitely false”) to 4 (“definitely true”). Scores for each subscale range from 4 to 16 points, while the total hope score, calculated as the sum of the two subscales, ranges from 8 to 32 points. Higher scores indicate a higher level of dispositional hope [23].

#### 2.1.3. Ad Hoc Questionnaire of Accompanying Emotions

To assess the emotional experiences accompanying lung cancer, a researcher-developed ad hoc questionnaire was used. The questionnaire comprised 14 negative emotional states: anger, helplessness/powerlessness, grief/regret, sadness/dejection, disgust, shame, embarrassment, sense of injustice, despair/crying, emptiness, sense of threat, anxiety, fear, pessimism. The selection of emotional states was informed by the broader literature on psychological responses to cancer, including dimensions of helplessness/hopelessness and anxious preoccupation described in the Mental Adjustment to Cancer framework, as well as emotional and psychosocial problems identified in cancer-related distress assessment [4,24,25,26].

For each emotional state, participants rated its intensity on a 5-point scale ranging from 1 (“minimal intensity”) to 5 (“maximum intensity”). For the purposes of the cluster analysis, responses were coded as 1 when an emotion was indicated and 0 when no response was recorded. The questionnaire was developed for the purposes of the present study and was not a standardized psychometric instrument.

#### 2.1.4. Sociodemographic and Temperamental Datasheet

A researcher-developed datasheet was used to collect basic sociodemographic information, including gender, age, marital status/cohabitation, and subjective assessment of financial situation. The datasheet also included two categorisation questions concerning temperamental characteristics, used as supplementary variables rather than as comprehensive, standardized psychometric measures of temperament. For basic mood, participants selected one of four categories: cheerful–stable, sad–stable, cheerful–unstable, or sad–unstable. For overall pace of activity and life dynamics, participants selected one of four categories: slow–active, fast–active, slow–passive, or fast–passive. Participants were instructed to select the category that best described their usual characteristics. The use of brief categorical questions was intended to minimize the assessment burden in patients with lung cancer, for whom extensive assessment procedures may be particularly demanding due to illness-related physical and psychological distress [19,27].

### 2.2. Study Participants (Sample Characteristics)

The study included 104 patients with a clinically confirmed diagnosis of lung cancer. Of the 115 patients considered for inclusion, 11 were excluded because their health condition prevented them from completing the study materials. Consecutive sampling was employed, with eligible patients presenting at the study center approached sequentially for participation. The gender distribution was relatively balanced, with a slight predominance of men (52.88%, *N* = 55), while women accounted for 47.12% (*N* = 49).

The majority of participants were older adults. The largest age group comprised individuals aged 65–75 years (44.23%, *N* = 46), followed by those aged 40–65 years (26.92%, *N* = 28). Participants aged up to 40 years accounted for 15.39% (*N* = 16), whereas those over 75 years constituted 13.46% (*N* = 14). Most participants lived with their family (80.77%, *N* = 84), while 19.23% (*N* = 20) lived alone. Regarding financial situation, 54.81% (*N* = 57) of the participants assessed it as good and 7.69% (*N* = 8) as very good. A total of 31.73% (*N* = 33) assessed their financial situation as average, while 5.77% (*N* = 6) considered it poor.

Temperamental characteristics were assessed using a self-report approach. Participants were presented with several predefined categories of basic mood and overall pace of activity and selected the category that they considered most representative of their usual characteristics. Regarding basic mood, the most common categories were cheerful–stable (39.42%, *N* = 41) and sad–stable (32.69%, *N* = 34). A cheerful–unstable mood was reported by 20.19% (*N* = 21) of participants, while a sad–unstable mood was reported by 7.69% (*N* = 8). In terms of overall pace of activity, the largest proportion of participants was characterised by a slow–active pace (44.23%, *N* = 46), followed by a fast–active pace (25.96%, *N* = 27). The remaining participants were characterised by a slow–passive pace (17.31%, *N* = 18) or a fast–passive pace (12.50%, *N* = 13).

### 2.3. Study Procedure and Ethical Considerations

The study was conducted through face-to-face contact with patients hospitalized in the Tuberculosis and Lung Diseases Ward of the Department of Pulmonary Diseases at the Stefan Cardinal Wyszyński Provincial Specialist Hospital in Lublin, Poland. Participation was voluntary and anonymous. Before participation, patients were informed about the scientific purpose of the study, the nature of their participation, and their right to withdraw at any stage without consequences. Written informed consent was obtained from all participants prior to completion of the research materials using the paper-and-pencil method (Paper-and-Pencil Interview, PAPI). The collected data were coded and analysed in an anonymised form, with results reported only at the group level.

Participants were eligible for inclusion if they were at least 18 years of age, had a confirmed diagnosis of lung cancer, provided informed consent, and were able to understand the study instructions and complete the questionnaires independently. Patients who did not meet these criteria, including those who declined participation or were unable to complete the research materials because of impaired cognitive functioning, were not included in the study.

The study was conducted between 1 July 2024 and 31 December 2024. Ethical approval was obtained from the Bioethics Committee of the Medical University of Lublin (Annex to Order No. 148/2023 of the Rector of the Medical University of Lublin, dated 24 November 2023; approval No. KE/503/06/2024).

### 2.4. Statistical Analysis Methods

Statistical analyses were performed using the Statistica 14.1.0.4 software (Cloud Software Group, Inc., 2023). Descriptive statistics, including arithmetic means (*M*), standard deviations (*SD*), medians (*Me*), quartiles (*Q*_1_, *Q*_3_), modes (*Mo*) and minimum (*Min*) and maximum (*Max*) values, were used to describe the study sample and the distributions of continuous variables, including the AIS and ADHS scores. Categorical variables were presented using frequencies (*N*) and percentages (%).

Spearman’s correlation coefficient (*ρ*) was used to assess the direction and strength of associations between continuous variables, including the intensity ratings of individual emotions, the total ADHS score and its subscale scores, and the AIS score. The non-parametric Kruskal–Wallis H test was used to examine whether basic mood and overall pace of activity differentiated levels of disease acceptance and hope across multiple categories.

Exploratory hierarchical agglomerative cluster analysis was performed to identify subgroups of patients based on the configuration of 14 dichotomous indicators representing the presence or absence of individual negative emotions. The unweighted pair-group method with arithmetic mean (UPGMA) was used, with percentage mismatch as the dissimilarity measure. This measure expresses dissimilarity as the proportion of indicators for which two participants have different responses. Consequently, both joint presence and joint absence of an emotion contribute equally to similarity between participants.

The two-cluster solution was selected based on inspection of the dendrogram and the interpretability of the resulting groups. A three-cluster solution yielded a cluster containing only two participants, which limited its interpretability. To assess the stability of the selected solution, a subsampling analysis was performed using the same clustering algorithm and dissimilarity measure. One thousand random subsamples, each comprising 80% of the original sample, were analysed. The resulting partitions were compared with the original partition using the adjusted Rand index (ARI). As a sensitivity analysis, the clustering procedure was repeated using Jaccard distance to examine the extent to which the identified solution depended on the selected dissimilarity measure.

Following the identification of the two clusters, the Mann–Whitney U test was used to compare levels of disease acceptance (AIS) and hope (ADHS) between the groups. The Mann–Whitney U test was also used to compare the total number of endorsed negative emotions between the two clusters. Effect size for the comparisons of AIS and hope was estimated using the r coefficient, whereas rank-biserial correlation was used to quantify the effect size for the comparison of the number of endorsed emotions. The chi-square (*χ*^2^) test was used to examine differences in categorical sociodemographic variables, including gender and financial situation, as well as temperamental characteristics, between the identified clusters.

To account for multiple testing, the Benjamini–Hochberg procedure was applied to the 56 correlations reported in the study to control the false discovery rate, using an adjusted p-value threshold of 0.05. The Holm procedure was applied to the six comparisons of demographic and self-reported characteristics between clusters and separately to the four comparisons of disease acceptance and hope outcomes between clusters. For the comparisons of basic mood and overall pace of activity with disease acceptance and hope, all unadjusted *p*-values exceeded 0.05.

For all statistical analyses, statistical significance was set at *p* < 0.05.

## 3. Results

### 3.1. Profile and Intensity of Negative Emotions Experienced by Patients

First, the occurrence and intensity of 14 negative emotional states were evaluated in the study group (*N* = 104). The results showed considerable variation in the frequency with which individual emotional states were endorsed, with anger and helplessness/powerlessness being the most frequently indicated.

Anger was the most frequently indicated emotional state (57.69%), with 31.73% of the total sample assigning it the highest intensity rating of 5 on a 1–5 scale. Helplessness/powerlessness was the second-most frequently indicated emotional state (40.38%), including 18.27% of patients who assigned it an intensity of 5. Sadness/dejection (32.69%) and anxiety (31.73%) were also indicated by more than 30% of the respondents, while grief/regret was indicated by 30.77% of participants. Disgust and emptiness were the least frequently endorsed emotional states, each occurring in 21.15% of the sample. For shame, embarrassment, and disgust, the most common response was the lowest intensity rating (1). The detailed distribution of the frequency and intensity of individual emotional states is presented in Table 1.

### 3.2. Level of Disease Acceptance and Hope

The level of disease acceptance and hope was assessed using the Acceptance of Illness Scale (AIS) and Snyder’s Adult Dispositional Hope Scale (ADHS) in the study group (*N* = 104). The mean AIS score was 23.14 ± 8.29 points (*Me* = 23.0). Based on the AIS categories used in the present study, 27.88% (*N* = 29) of the patients had a low level of disease acceptance, 65.39% (*N* = 68) had a moderate level, and 6.73% (*N* = 7) had a high level of disease acceptance [22]. These findings are consistent with previous Polish research, in which patients with lung cancer had the lowest mean AIS score (23.17) among the cancer groups examined, including breast, colorectal, and prostate cancer [6]. Detailed descriptive statistics for disease acceptance and hope are presented in Table 2.

In contrast, the mean overall hope score was 25.89 ± 5.21 points (*Me* = 27.00) on a scale ranging from 8 to 32 points. The mean scores for the two components of hope were similar: 12.88 ± 2.78 points for the Agency subscale and 13.02 ± 2.76 points for the Pathways subscale. Both subscale means were above the theoretical midpoint of their respective scales.

### 3.3. Correlational Relationships Between Emotions, Disease Acceptance, and Hope

The correlation analysis revealed several nominally significant associations between the intensity of reported negative emotions and indicators of disease acceptance and hope before adjustment for multiple testing. A negative correlation was observed between the intensity of anger and the overall AIS score (*ρ* = −0.282, *p* = 0.029). In addition, the intensity of embarrassment was negatively correlated with the Agency subscale of the ADHS (*ρ* = −0.530, *p* = 0.008) and the overall hope score (*ρ* = −0.410, *p* = 0.047). However, none of these associations remained statistically significant after applying the Benjamini–Hochberg false discovery rate correction across the 56 correlations. These findings should therefore be interpreted as exploratory. The nominally significant correlations, together with the corresponding unadjusted *p*-values, FDR-adjusted *q*-values, and 95% confidence intervals, are presented in Table 3; the complete set of 56 correlations is provided in Supplementary Table S1.

### 3.4. Association of Demographic and Temperamental Characteristics with Disease Acceptance and Hope

To examine whether selected demographic and self-reported temperamental characteristics were associated with disease acceptance and hope, non-parametric comparisons were performed using the Kruskal–Wallis H test. Neither basic mood nor overall pace of activity was significantly associated with AIS scores or any of the hope measures (Agency, Pathways, or overall hope; all *p* > 0.05).

### 3.5. Exploratory Cluster Analysis of Reported Negative Emotions

Due to the limited number of statistically significant associations identified in the correlational and comparative analyses, an exploratory cluster analysis was performed to examine whether subgroups of patients with different configurations of endorsed negative emotions could be identified. Hierarchical agglomerative clustering was conducted using the unweighted pair-group method with arithmetic mean (UPGMA) and percentage mismatch as the dissimilarity measure. The analysis included 14 dichotomous variables indicating the presence or absence of individual emotions (0 = no, 1 = yes).

Inspection of the dendrogram initially indicated the possibility of a three-cluster solution. However, one of the clusters comprised only two patients. Due to its small size and limited interpretability, a two-cluster solution was ultimately adopted. The dendrogram and the linkage distance across agglomeration steps are provided in Supplementary Figures S1 and S2, respectively. The analysis identified two exploratory clusters that differed primarily in the number of endorsed negative emotions.

Cluster 1 (75.0% of participants; *N* = 78) comprised patients endorsing a relatively small number of negative emotions. The most frequently endorsed emotions in this cluster were anger (44.9%), helplessness/powerlessness (25.6%), and sadness/dejection (14.1%), while the remaining emotions were endorsed by less than 12% of patients.

Cluster 2 (25.0% of participants; *N* = 26) comprised patients endorsing a broader range of negative emotions. Particularly high frequencies were observed for sense of injustice (100.0%), grief/regret (96.2%), anger (96.2%), anxiety (92.3%), sadness/dejection (88.5%), shame (88.5%), embarrassment (88.5%), fear (88.5%), and pessimism (88.5%). Helplessness/powerlessness, disgust, despair/crying, emptiness, and sense of threat were each endorsed by 84.6% of patients in this cluster.

To further assess whether the cluster solution provided information beyond the simple number of endorsed emotions, the total number of endorsed negative emotions was compared between the two clusters. Cluster 1 comprised patients who endorsed a mean of 1.28 emotions (*SD* = 0.75; median = 1; range = 1–5), whereas Cluster 2 comprised patients who endorsed a mean of 12.50 emotions (*SD* = 1.98; median = 14; range = 8–14). The clusters differed significantly in the number of endorsed emotions (Mann–Whitney *U* = 0.00, *Z* = −8.71, *p* < 0.001, rank-biserial correlation = 1.00).

Overall, the two clusters differed primarily in the number and breadth of endorsed negative emotions rather than in a specific configuration of individual emotions. The distribution of individual emotions across the two clusters is presented in Table 4.

The subsampling analysis indicated high stability of the two-cluster solution under the original clustering procedure. Across 1000 subsamples, each containing 80% of the participants, the partition was identical to the original partition for the participants included in each subsample (ARI = 1.00 in all comparisons). However, a sensitivity analysis using Jaccard distance yielded a substantially different partition, consisting of one cluster containing 103 participants and another containing one participant. These findings indicate that the original solution was stable under subsampling when the same dissimilarity measure was used, but was sensitive to the choice of dissimilarity measure. Therefore, the identified clusters should be interpreted as exploratory and primarily as reflecting differences in the number and breadth of endorsed negative emotions rather than distinct emotional profiles.

### 3.6. Demographic and Temperamental Differences Between Clusters

The demographic and self-reported characteristics of patients in the two clusters were compared using chi-square tests. Before adjustment for multiple testing, nominally significant differences were observed between the clusters in gender (χ^2^(1) = 4.644, *p* = 0.031) and financial situation (χ^2^(1) = 6.031, *p* = 0.014). Women constituted a larger proportion of Cluster 2 (65.38%, *N* = 17) than of Cluster 1 (41.03%, *N* = 32), whereas men constituted a larger proportion of Cluster 1 (58.97%, *N* = 46) than of Cluster 2 (34.62%, *N* = 9). Regarding financial situation, patients in Cluster 1 more frequently reported a very good or good financial situation (69.23%, *N* = 54), whereas a moderate or poor financial situation was more common in Cluster 2 (57.69%, *N* = 15). However, neither difference remained statistically significant after adjustment for multiple testing using the Holm procedure.

No statistically significant differences were found in age, living arrangements, basic mood, or overall pace of activity before adjustment (all *p* > 0.05). Detailed results are presented in Table 5.

### 3.7. Disease Acceptance and Hope by Cluster

Disease acceptance and hope were compared between the two identified clusters using the Mann–Whitney U test. A statistically significant difference in disease acceptance (AIS) was observed between the clusters (*Z* = −2.879, *p* = 0.004, *r* = 0.28), with patients in Cluster 2 showing higher AIS scores than those in Cluster 1. The mean AIS score was 27.19 ± 6.89 (*Me* = 27.0) in Cluster 2 and 21.79 ± 8.31 (*Me* = 21.0) in Cluster 1. This difference remained statistically significant after adjustment for multiple testing using the Holm procedure. No statistically significant differences were found between the clusters in the Agency subscale of the ADHS (*Z* = 0.863, *p* = 0.388), the Pathways subscale (*Z* = 0.180, *p* = 0.857), or the overall hope score (*Z* = 0.548, *p* = 0.584). The median overall hope score was 27.0 in both clusters. Detailed descriptive statistics and Mann–Whitney U test results are presented in Table 6.

## 4. Discussion

The present study aimed to explore the relationships between sociodemographic and temperamental characteristics, negative emotional experiences, hope, and disease acceptance in a group of 104 patients with lung cancer. The most notable finding was the identification of two patient clusters characterised by different numbers of endorsed negative emotional states. Cluster 2, comprising 25% of the study sample, was characterised by a broader range of endorsed negative emotions and, at the same time, had significantly higher disease acceptance scores than Cluster 1, which comprised 75% of the participants and was characterised by a smaller number of endorsed negative emotions (AIS: 27.19 vs. 21.79, respectively; *p* = 0.004).

This finding suggests that the experience of negative emotions and acceptance of illness may not necessarily represent opposite dimensions of psychological adaptation in patients with lung cancer. Rather than indicating that greater acceptance is accompanied by an absence of negative affect, the present results point to a potentially more complex relationship between negative emotional experiences and adaptation to illness. Such a pattern is consistent with theoretical perspectives suggesting that psychological adjustment following a potentially traumatic experience may be accompanied by the simultaneous presence of distress and adaptive processes [28,29,30,31]. However, the cross-sectional and exploratory nature of the present study does not allow conclusions to be drawn about the mechanisms underlying this association.

### 4.1. Interpreting the Relationship Between Negative Emotions and Disease Acceptance

The finding that Cluster 2 was characterised by a broader range of endorsed negative emotional states with higher disease acceptance may be considered in light of theoretical models of cognitive adaptation to traumatic experiences. According to Janoff-Bulman’s theory of assumptive world and schema change, a life-threatening event may challenge fundamental beliefs concerning safety, predictability, control, and justice [31,32,33]. The process of adapting to such an experience may be associated with discrepancies between previous assumptions and the new reality, which may be accompanied by substantial emotional distress and cognitive processing of the event [31,34,35].

From this perspective, the coexistence of a broader range of endorsed negative emotional experiences and higher disease acceptance observed in Cluster 2 does not necessarily indicate an inconsistency between emotional distress and psychological adaptation. Rather, it may suggest that these dimensions can coexist during adaptation to a serious illness. Patients may experience anger, regret, anxiety, or a sense of injustice while simultaneously acknowledging the reality of their illness and its consequences. The present findings are therefore compatible with theoretical perspectives emphasising that distress and adaptive cognitive processes do not necessarily occur as mutually exclusive states [30,31]. One possible hypothesis is that acknowledging a broader range of negative emotions may reflect greater engagement with the emotional experience of illness, which may coexist with greater disease acceptance. However, this interpretation remains speculative, as the number of reported emotions does not directly indicate the extent or nature of cognitive processing.

A complementary interpretation can be derived from the two-component model of posttraumatic growth proposed by Maercker and Zoellner [36], which distinguishes constructive cognitive processing from more illusory aspects of perceived growth. Importantly, however, the present study did not directly assess posttraumatic growth, cognitive processing, or repressive coping. Therefore, these theoretical frameworks should be regarded as possible explanations for the observed pattern rather than mechanisms demonstrated by the present data. Furthermore, the cross-sectional design does not allow us to determine whether greater emotional engagement contributes to disease acceptance, whether disease acceptance influences the reporting of emotions, or whether both are associated with other factors. Longitudinal studies using direct measures of cognitive and emotional processing are needed to examine these possibilities.

The lower number of endorsed negative emotional states observed in Cluster 1, together with its lower level of disease acceptance, also warrants consideration. One possible interpretation is that a lower reported number of negative emotions does not necessarily reflect better psychological adaptation. Previous research has shown that patients with lung cancer may differ in their psychological profiles, and that emotional responses and adjustment cannot be reduced to a single dimension of distress [11]. However, the present study did not assess emotional avoidance or repression directly; therefore, it would be inappropriate to classify Cluster 1 as a repressive coping group. The observed pattern instead suggests that the relationship between emotional experience and disease acceptance may be more complex than a simple inverse association.

### 4.2. The Role of Stigmatisation, Gender, and Financial Situation in Lung Cancer

The broader range of endorsed negative emotional states observed in Cluster 2 may also be considered in the context of the specific psychosocial challenges associated with lung cancer. Lung cancer is a disease frequently affected by stigma, particularly because of its strong social association with tobacco smoking. Previous studies have shown that patients with lung cancer may experience stigma, guilt, shame, anxiety, and social rejection, with these experiences potentially affecting psychological well-being and quality of life [37,38,39,40].

The particularly high number of endorsements of a sense of injustice (100%), anger (96.2%), anxiety (92.3%), and shame and embarrassment (88.5% each) observed in Cluster 2 is therefore noteworthy. These findings may be compatible with the emotional consequences of perceived stigma and the social meanings attached to lung cancer. However, the present study did not directly assess experiences of stigma, perceived blame, or stigma resistance. Consequently, the observed emotional pattern cannot be attributed specifically to stigmatisation, although stigma represents one plausible context for understanding these findings.

The demographic composition of the clusters also provides an important context for interpreting the results. Women constituted a larger proportion of Cluster 2 than of Cluster 1 (65.38% vs. 41.03%), while men predominated in Cluster 1. Previous research has reported differences in psychological distress following cancer diagnosis according to gender, including higher rates of anxiety and depression among women in some cancer populations [41]. In the present study, however, the cross-sectional design does not allow the observed gender difference to be interpreted as a causal explanation for the greater number of endorsed negative emotional states in Cluster 2.

A similar pattern was observed for financial situation. Patients in Cluster 2 more frequently reported an average or poor financial situation than those in Cluster 1 (57.69% vs. 30.77%). Financial circumstances may constitute an important contextual factor in psychological adjustment to cancer, as economic difficulties can increase the burden associated with treatment and illness-related uncertainty. Recent research among patients with lung cancer has also highlighted the relationship between psychosocial stressors and psychological distress [9]. Nevertheless, because the present study assessed subjective financial situation rather than treatment-related financial toxicity, the findings should not be interpreted as direct evidence of financial toxicity.

Taken together, the results indicate that gender and subjective financial situation differed descriptively between the two clusters, although these differences did not remain statistically significant after adjustment for multiple testing. These factors may form part of the broader psychosocial context in which patients experience and adapt to lung cancer, but further research is needed to determine the mechanisms underlying these associations.

### 4.3. Hope and Negative Emotional Experiences

Another important finding of the present study was the absence of statistically significant differences in dispositional hope between the two identified clusters. Neither the Agency subscale, the Pathways subscale, nor the overall hope score differed significantly between the groups. The median overall hope score was 27.0 points in both clusters, despite the substantial differences in the number of endorsed negative emotional states.

This finding can be considered in the context of Snyder’s cognitive theory of hope, which conceptualises hope as goal-directed thinking involving two interrelated components: agency, referring to the perceived capacity to initiate and sustain movement toward goals, and pathways, referring to the perceived ability to identify alternative routes toward those goals [12,23]. The absence of significant differences in these dimensions across the two emotional clusters suggests that dispositional hope may not be directly determined by the number of endorsed negative emotional experiences. Rather, hope and emotional distress may represent partially distinct dimensions of psychological functioning.

This interpretation is consistent with research conducted in patients with lung cancer. Berendes et al. [13] found that hope was associated with psychological adjustment and symptom-related outcomes, while also demonstrating that the presence of hope did not imply an absence of distress. Thus, patients may experience anxiety, pain, or other negative emotional states while retaining goal-directed thinking and the capacity to identify meaningful goals. Similar perspectives have been proposed in the broader literature on hope and adjustment to chronic illness, where hope may support the reformulation of goals and the identification of alternative pathways when previously established goals become difficult to attain [42].

It is also important to recognize that hope in clinical settings may extend beyond an individual psychological resource. Hope may be dynamic and relational and may be supported through patient–clinician interactions, clarification of meaningful goals, and identification of realistic pathways toward desired outcomes [43]. Mylod and Lee [44] have emphasized the role of clinicians in supporting hope by clarifying patient-centred goals, demonstrating commitment and the ability to help, and identifying realistic pathways toward meaningful outcomes. The present study assessed hope using Snyder’s Hope Scale and therefore primarily captured its individual cognitive dimension; it did not assess the relational or therapeutic dimensions of hope.

The present findings therefore suggest that the number of endorsed negative emotional experiences associated with lung cancer and dispositional hope, as assessed by Snyder’s Hope Scale, should not necessarily be regarded as opposite dimensions. In particular, the comparable levels of hope observed in both clusters indicate that the endorsement of a broader range of negative emotions does not necessarily coincide with lower agency, lower pathways thinking, or lower overall dispositional hope. However, the cross-sectional design of the study does not allow conclusions to be drawn about the stability of hope over time or about causal relationships between hope and emotional experiences.

### 4.4. Summary and Clinical Implications

The findings of the present study may have several implications for psycho-oncological practice. First, a smaller number of endorsed negative emotional states should not automatically be interpreted as evidence of better psychological adaptation. Patients in Cluster 1 reported fewer negative emotional states but also had lower disease acceptance than patients in Cluster 2. This finding suggests that the number of endorsed negative emotions and disease acceptance should be assessed separately rather than assuming that a lower number of reported negative emotions necessarily reflects better adaptation. Although emotional avoidance or repressive coping may represent possible explanations for such a pattern, these processes were not directly assessed in the present study and therefore require further investigation [11,45].

Second, the findings suggest that the endorsement of negative emotions such as anger, anxiety, or a sense of injustice should be considered in the broader context of the patient’s psychological adaptation rather than interpreted in isolation. In clinical practice, exploring such emotional experiences may provide an opportunity to explore patients’ experiences of illness, perceived stigma, concerns, and individual meanings attributed to the diagnosis. Acceptance-focused cognitive behavioural approaches and interventions addressing lung cancer stigma may be relevant in this context [40,46].

Third, the absence of significant differences in dispositional hope between the two clusters suggests that hope may constitute a psychological dimension that is not directly reflected by the number of endorsed negative emotional experiences. From a clinical perspective, assessment of hope may therefore provide information complementary to the assessment of emotional distress and disease acceptance. Previous research has also highlighted the relevance of patient–provider communication in the context of lung cancer stigma and psychological well-being [47].

Overall, the results support the value of a multidimensional psycho-oncological assessment in patients with lung cancer, encompassing emotional experiences, disease acceptance, and hope rather than relying on any single indicator of psychological adjustment. Such an approach may help identify patients whose emotional experiences, disease acceptance, and hope present different patterns, although its potential value for guiding supportive care requires further investigation. Given the exploratory and cross-sectional nature of the present study, these findings should be considered hypotheses-generating and require confirmation in longitudinal and larger-scale studies.

### 4.5. Limitations of the Study

Several limitations should be considered when interpreting the findings. First, the cross-sectional design does not allow conclusions to be drawn about causal relationships or changes in disease acceptance, hope, and emotional experiences over time. Second, emotional experiences were assessed using a researcher-developed, ad hoc questionnaire rather than a standardized and psychometrically validated measure. Although this approach allowed the assessment of a broad range of emotions relevant to the study, it limits the comparability of the findings with studies using validated instruments. In addition, the wording of the emotion item did not explicitly instruct participants that multiple emotions could be selected. Consequently, an unrecorded response may reflect either non-endorsement of a given emotion or the participant’s interpretation of the response format. This issue may have influenced the observed distribution of the number of endorsed emotions and should be considered when interpreting the cluster analysis. Third, the assessment of emotions was based on self-report and may therefore have been influenced by recall, reporting, or social desirability biases.

The study included a relatively small sample of 104 patients recruited using consecutive sampling from a single clinical setting, which may limit the generalisability of the findings to the broader population of patients with lung cancer. An additional limitation concerns the variables labelled as temperamental characteristics, which were assessed using single-item categorical questions developed by the researchers rather than standardized, psychometrically validated measures of temperament. Consequently, their reliability and validity have not been established, and findings involving these variables should be interpreted with caution. Future studies should use validated instruments to assess temperament. The study also did not systematically collect potentially important clinical variables, such as cancer stage, treatment response, performance status, and smoking history, which may have influenced patients’ emotional experiences, disease acceptance, and hope.

Furthermore, depression and anxiety were not assessed using validated measures, and their potential influence on the observed associations between disease acceptance, hope, and quality of life cannot be ruled out. Finally, the cluster analysis was exploratory, and the identified two-cluster solution should be considered hypothesis-generating rather than definitive. Although the solution showed high subsampling stability when the same clustering procedure was retained, the results were sensitive to the choice of dissimilarity measure. Replication in larger, multicentre samples using validated measures of emotional distress and temperament, comprehensive clinical data, and longitudinal designs is warranted. Given that the clusters differed primarily in the number and breadth of endorsed negative emotions, rather than representing clearly distinct emotional profiles, their clinical interpretation should therefore remain cautious.

## 5. Conclusions

The present exploratory study suggests that patients with lung cancer who reported a greater number and broader range of negative emotional experiences had higher disease acceptance scores, while dispositional hope did not differ significantly between the identified exploratory clusters. Disease acceptance and hope were also differentially associated with specific emotional experiences, whereas basic mood and overall pace of activity were not significantly associated with these outcomes. However, the interpretation of the identified exploratory clusters remains uncertain because of the unusual distribution of reported emotions, the researcher-developed nature of the emotion measure, and the sensitivity of the clustering results to the selected dissimilarity measure. Therefore, the observed association between a greater number of endorsed negative emotional experiences and higher disease acceptance should be considered exploratory and requires confirmation in larger, longitudinal studies using validated measures. Overall, the findings suggest that psychological adaptation to lung cancer should not be understood solely in terms of the absence of negative emotions.

### Recommendations

Psycho-oncological care for patients with lung cancer may benefit from multidimensional assessment that considers emotional experiences, disease acceptance, and hope alongside sociodemographic circumstances. Particular attention may be given to patients reporting a broader range of negative emotional experiences, difficulties with disease acceptance, or a poorer subjective financial situation, while avoiding the assumption that a lower number of reported negative emotions necessarily indicates better adaptation. Further research should test the reproducibility of the exploratory clustering solution in larger and multicentre samples, use validated measures of emotional distress, and employ longitudinal designs to clarify the temporal and potentially causal relationships between emotional experiences, disease acceptance, and hope.

## Figures and Tables

**Table 1:** Emotions Experienced by Patients with Lung Cancer and Their Intensity.

Emotions	Intensity of Experienced Emotions (1—Low, 5—High)										Total
	1		2		3		4		5		
	*N*	%	*N*	%	*N*	%	*N*	%	*N*	%	%
anger	8	7.69	6	5.77	11	10.58	2	1.92	33	31.73	57.69
helplessness/powerlessness	2	1.92	7	6.73	8	7.69	6	5.77	19	18.27	40.38
grief/regret	6	5.77	4	3.85	7	6.73	4	3.85	11	10.58	30.77
sadness/dejection	6	5.77	2	1.92	10	9.62	7	6.73	9	8.65	32.69
disgust	13	12.50	3	2.88	4	3.85	0	0.0	2	1.92	21.15
shame	13	12.50	5	4.81	2	1.92	1	0.96	3	2.88	23.08
embarrassment	13	12.50	5	4.81	2	1.92	1	0.96	3	2.88	23.08
sense of injustice	10	9.62	4	3.85	3	2.88	2	1.92	8	7.69	25.96
despair/crying	5	4.81	5	4.81	5	4.81	4	3.85	4	3.85	22.12
emptiness	4	3.85	2	1.92	7	6.73	5	4.81	4	3.85	21.15
sense of threat	5	4.81	3	2.88	2	1.92	5	4.81	11	10.58	25.0
anxiety	5	4.81	2	1.92	3	2.88	7	6.73	16	15.38	31.73
fear	7	6.73	3	2.88	1	0.96	4	3.85	14	13.46	27.88
pessimism	7	6.73	8	7.69	0	0.0	3	2.88	9	8.65	25.96

Note: Percentages for individual intensity levels are calculated based on the total study sample (*N* = 104). The total *N* and % represent the number and percentage of participants who reported experiencing a given emotion.

**Table 2:** Descriptive Statistics of Disease Acceptance and Hope.

Scale	Descriptive Statistics (*N* = 104)								
	*M*	*SD*	*Me*	*Q*_1_	*Q*_3_	*Min*	*Max*	*Mo*	*Mo* (*N*)
Disease Acceptance (AIS)	23.14	8.29	23.0	18.0	29.0	8.0	40.0	20.0	12
Hope—Agency Subscale	12.88	2.78	13.0	12.0	15.0	4.0	16.0	16.0	23
Hope—Pathways Subscale	13.02	2.76	14.0	12.0	15.0	5.0	16.0	16.0	22
Hope—Overall Score	25.89	5.21	27.0	24.0	30.0	11.0	32.0	32.0	18

**Table 3:** Nominally Significant Correlations Between Reported Negative Emotions, Disease Acceptance, and Hope.

**Emotional State**	**Outcome**	***N***	***ρ***	***p***	***q*** ** (BH-FDR)**	**95% *CI* for *ρ***
Anger	Disease acceptance (AIS)	60	−0.282	0.029	0.457	[−0.509, −0.037]
Embarrassment	Hope—Agency	24	−0.530	0.008	0.224	[−0.490, 0.238]
Embarrassment	Hope—Overall	24	−0.410	0.047	0.457	[−0.496, 0.397]

Note: *ρ* = Spearman’s rank correlation coefficient; *p* = unadjusted *p*-value; *q* = Benjamini–Hochberg false discovery rate-adjusted *p*-value; *N* = number of participants reporting the respective emotional state; 95% *CI* = 95% bootstrap confidence interval for Spearman’s *ρ*, based on 5000 bootstrap replications. None of the 56 correlations remained statistically significant after false discovery rate correction.

**Table 4:** Frequency of Endorsed Emotions in the Identified Patient Clusters.

Emotions	Cluster 1 (*N* = 78)	Cluster 2 (*N* = 26)
anger	44.9%	96.2%
helplessness/powerlessness	25.6%	84.6%
grief/regret	9.0%	96.2%
sadness/dejection	14.1%	88.5%
disgust	0.0%	84.6%
shame	1.3%	88.5%
embarrassment	1.3%	88.5%
sense of injustice	1.3%	100.0%
despair/crying	1.3%	84.6%
emptiness	0.0%	84.6%
sense of threat	5.1%	84.6%
anxiety	11.5%	92.3%
fear	7.7%	88.5%
pessimism	5.1%	88.5%

**Table 5:** Demographic and Temperamental Characteristics of Patients in the Identified Clusters.

Variable	Category	Cluster 1 (*N* = 78)	Cluster 2 (*N* = 26)
Gender	female	32 (41.03%)	17 (65.38%)
	male	46 (58.97%)	9 (34.62%)
Age	up to 40 years	12 (15.38%)	4 (15.38%)
	40–65 years	18 (23.08%)	10 (38.46%)
	65–75 years	35 (44.87%)	11 (42.31%)
	over 75 years	13 (16.67%)	1 (3.85%)
Living arrangements	alone	17 (21.79%)	3 (11.54%)
	with family	61 (78.21%)	23 (88.46%)
Financial situation	very good, good	54 (69.23%)	11 (42.31%)
	average, poor	24 (30.77%)	15 (57.69%)
Basic mood	sad–unstable	4 (5.13%)	4 (15.38%)
	sad–stable	29 (37.18%)	5 (19.23%)
	cheerful–unstable	16 (20.51%)	5 (19.23%)
	cheerful–stable	29 (37.18%)	12 (46.15%)
Overall pace of activity	slow–passive	12 (15.38%)	6 (23.08%)
	slow–active	34 (43.59%)	12 (46.15%)
	fast–passive	10 (12.82%)	3 (11.54%)
	fast–active	22 (28.21%)	5 (19.23%)

**Table 6:** Disease Acceptance and Hope Scores by Patient Cluster.

Scale	Cluster 1 (*N* = 78)					Cluster 2 (*N* = 26)					Mann–Whitney U Test	
	*M*	*SD*	*Me*	*Q*_1_	*Q*_3_	*M*	*SD*	*Me*	*Q*_1_	*Q*_3_	*Z*	*p*
Disease Acceptance (AIS)	21.79	8.31	21.0	16.0	28.0	27.19	6.89	27.0	24.0	32.0	−2.879	0.004
Hope—Agency Subscale	13.04	2.68	13.0	12.0	15.0	12.38	3.03	13.0	11.0	14.0	0.863	0.388
Hope—Pathways Subscale	12.97	2.90	13.5	12.0	15.0	13.15	2.31	14.0	11.0	15.0	0.180	0.857
Hope—Overall Score	26.01	5.34	27.0	24.0	30.0	25.54	4.87	27.0	23.0	29.0	0.548	0.584

Note. *p*-values are unadjusted. The Holm procedure was applied to adjust for multiple comparisons across the four outcomes; the AIS difference remained statistically significant after adjustment.

## Data Availability

The original contributions presented in this study are included in the supplementary material. Further inquiries can be directed to the corresponding author.

## Supplementary Materials

The following supporting information can be downloaded at https://www.mdpi.com/article/10.3390/cancers18213575/s1. Dataset corrected.xlsx. Figure S1: Tree Diagram. Figure S2: Linkage distance across agglomeration steps. Table S1: Spearman Correlations Between Reported Emotion Intensity and Disease Acceptance and Hope.

## Abbreviations

The following abbreviations are used in this manuscript:

AIS	Acceptance of Illness Scale
UPGMA	Unweighted Pair Group Method with Arithmetic Mean
ADHS	Adult Dispositional Hope Scale
