# Supplementary material for: Selected Demographic and Temperamental Characteristics of Patients with Lung Cancer and Their Associations with Disease Acceptance, Negative Emotional Experiences, and Hope
Source: Cancers (Basel). 2026 Nov 6;18(21):3575. doi: 10.3390/cancers18213575 (PMC13762058; doi:10.3390/cancers18213575)
Supplement: Supplementary file 1 [file cancers-18-03575-s001.zip › cancers-4687235-supplementary/Supplementary Files/Table S1.pdf]

Supplementary Table S1. Spearman Correlations Between Reported Emotion Intensity and Disease Acceptance and Hope.

Emotional state	Outcome	N	ϱ	p	q (BH-FDR)	95% CI for ϱ
Anger	Disease acceptance (AIS)	60	−0.282	0.029	0.457	[-0.509, -0.037]
Anger	Hope – Agency	60	0.042	0.614	0.932	[-0.541, 0.065]
Anger	Hope – Pathways	60	−0.013	0.902	0.962	[−0.482, 0.205]
Anger	Hope – Overall	60	0.007	0.946	0.962	[−0.588, 0.099]
Helplessness / powerlessness	Disease acceptance (AIS)	42	−0.253	0.085	0.595	[−0.618, 0.214]
Helplessness / powerlessness	Hope – Agency	42	−0.179	0.391	0.932	[−0.645, 0.091]
Helplessness / powerlessness	Hope – Pathways	42	−0.016	0.876	0.962	[−0.578, 0.274]
Helplessness / powerlessness	Hope – Overall	42	−0.118	0.254	0.932	[−0.270, 0.508]
Grief / regret	Disease acceptance (AIS)	32	−0.140	0.433	0.932	[−0.370, 0.485]
Grief / regret	Hope – Agency	32	−0.028	0.871	0.962	[−0.578, 0.182]
Grief / regret	Hope – Pathways	32	0.156	0.782	0.932	[−0.516, 0.243]
Grief / regret	Hope – Overall	32	0.067	0.715	0.932	[−0.571, 0.072]
Sadness / dejection	Disease acceptance (AIS)	34	−0.259	0.132	0.739	[−0.404, 0.346]
Sadness / dejection	Hope – Agency	34	−0.226	0.375	0.932	[−0.247, 0.402]
Sadness / dejection	Hope – Pathways	34	−0.194	0.465	0.932	[−0.207, 0.288]
Sadness / dejection	Hope – Overall	34	−0.272	0.151	0.769	[−0.475, 0.149]
Disgust	Disease acceptance (AIS)	22	−0.216	0.320	0.932	[−0.422, 0.373]
Disgust	Hope – Agency	22	−0.331	0.106	0.660	[−0.531, 0.113]
Disgust	Hope – Pathways	22	−0.008	0.962	0.962	[−0.661, 0.073]
Disgust	Hope – Overall	22	−0.220	0.300	0.932	[−0.718, 0.159]
Shame	Disease acceptance (AIS)	24	−0.320	0.047	0.457	[−0.774, −0.195]
Shame	Hope – Agency	24	−0.298	0.072	0.576	[−0.606, 0.220]
Shame	Hope – Pathways	24	0.019	0.862	0.962	[−0.578, 0.283]
Shame	Hope – Overall	24	−0.179	0.403	0.932	[−0.496, 0.397]
Embarrassment	Disease acceptance (AIS)	24	−0.161	0.543	0.932	[−0.552, 0.295]
Embarrassment	Hope – Agency	24	−0.530	0.008	0.224	[−0.490, 0.238]
Embarrassment	Hope – Pathways	24	−0.178	0.319	0.932	[−0.460, 0.314]
Embarrassment	Hope – Overall	24	−0.410	0.047	0.457	[−0.365, 0.403]
Sense of injustice	Disease acceptance (AIS)	27	0.129	0.522	0.932	[−0.249, 0.239]
Sense of injustice	Hope – Agency	27	−0.206	0.313	0.932	[−0.297, 0.284]

Sense of injustice	Hope – Pathways	27	−0.079	0.622	0.932	[−0.205, 0.502]
Sense of injustice	Hope – Overall	27	−0.140	0.488	0.932	[−0.506, 0.153]
Despair / crying	Disease acceptance (AIS)	23	0.075	0.709	0.932	[−0.454, 0.425]
Despair / crying	Hope – Agency	23	−0.172	0.415	0.932	[−0.437, 0.451]
Despair / crying	Hope – Pathways	23	0.107	0.616	0.932	[−0.551, 0.236]
Despair / crying	Hope – Overall	23	−0.043	0.837	0.962	[−0.472, 0.334]
Emptiness	Disease acceptance (AIS)	22	−0.237	0.606	0.932	[−0.292, 0.494]
Emptiness	Hope – Agency	22	−0.040	0.764	0.932	[−0.459, 0.512]
Emptiness	Hope – Pathways	22	0.031	0.751	0.932	[−0.484, 0.344]
Emptiness	Hope – Overall	22	−0.012	0.956	0.962	[−0.504, 0.223]
Sense of threat	Disease acceptance (AIS)	26	−0.141	0.495	0.932	[−0.454, 0.302]
Sense of threat	Hope – Agency	26	−0.142	0.514	0.932	[−0.314, 0.402]
Sense of threat	Hope – Pathways	26	−0.093	0.645	0.932	[−0.245, 0.257]
Sense of threat	Hope – Overall	26	−0.122	0.549	0.932	[−0.421, 0.207]
Anxiety	Disease acceptance (AIS)	33	−0.276	0.005	0.224	[−0.440, 0.373]
Anxiety	Hope – Agency	33	−0.144	0.527	0.932	[−0.578, 0.076]
Anxiety	Hope – Pathways	33	−0.155	0.614	0.932	[−0.625, 0.213]
Anxiety	Hope – Overall	33	−0.164	0.607	0.932	[−0.651, 0.286]
Fear	Disease acceptance (AIS)	29	−0.029	0.912	0.962	[−0.718, −0.032]
Fear	Hope – Agency	29	−0.086	0.750	0.932	[−0.558, 0.289]
Fear	Hope – Pathways	29	−0.087	0.734	0.932	[−0.440, 0.373]
Fear	Hope – Overall	29	−0.086	0.768	0.932	[−0.457, 0.434]
Pessimism	Disease acceptance (AIS)	27	0.116	0.049	0.457	[−0.548, 0.304]
Pessimism	Hope – Agency	27	0.033	0.751	0.932	[−0.524, 0.225]
Pessimism	Hope – Pathways	27	0.064	0.531	0.932	[−0.460, 0.314]
Pessimism	Hope – Overall	27	0.063	0.518	0.932	[−0.331, 0.418]

Note. ϱ = Spearman's rank correlation coefficient; p = unadjusted p–value; q = Benjamini–Hochberg false discovery rate–adjusted p–value; N = number of participants reporting the respective emotional state; 95% CI = 95% bootstrap confidence interval for Spearman's ϱ, based on 5,000 bootstrap replications. None of the 56 correlations remained statistically significant after false discovery rate correction.
